# Consistently High Frequency of Scooter Injuries in Children—Retrospective Data Analysis in a Level I Trauma Centre

**DOI:** 10.3390/children10091464

**Published:** 2023-08-28

**Authors:** Andrea Schuller, Anna Hohensteiner, Thomas Sator, Lorenz Pichler, Theresia Dangl, Cornelia Nass, Manuela Jaindl, Elisabeth Schwendenwein, Thomas M. Tiefenboeck, Stephan Payr

**Affiliations:** 1 University Clinic of Orthopaedics and Trauma Surgery, Department of Trauma Surgery, Medical University of Vienna, 1090 Vienna, Austria; andrea.schuller@meduniwien.ac.at (A.S.); anna.hohensteiner@meduniwien.ac.at (A.H.); thomas.sator@meduniwien.ac.at (T.S.); lorenz.pichler@meduniwien.ac.at (L.P.); theresia.dangl@meduniwien.ac.at (T.D.); cornelia.nass@meduniwien.ac.at (C.N.); manuela.jaindl@meduniwien.ac.at (M.J.); elisabeth.schwendenwein@meduniwien.ac.at (E.S.); thomas.tiefenboeck@meduniwien.ac.at (T.M.T.); 2 Section of Paediatric Trauma Surgery, Department of Trauma Surgery, University Clinic of Orthopaedics and Trauma Surgery, Medical University of Vienna, 1090 Vienna, Austria

**Keywords:** nonelectric scooter, paediatric injuries, head injuries, traffic accident

## Abstract

The aim of this retrospective study was to present an epidemiological overview of paediatric nonelectric-scooter-related injuries, focusing on changes in injury mechanism and frequency. A retrospective, descriptive data analysis at a Level I trauma centre, including patients aged from 0 to 18 years injured by riding nonelectric scooters, was performed. The observation period ranged from January 2015 to December 2022. The total study population consisted of 983 (mean age: 7.9 ± 4.0 years) children and adolescents, with most patients being male (800/983; 81.4%). The frequency of nonelectric scooter injuries was relatively consistent over the observation period. Patients sustained mostly minor injuries (lacerations, bone contusions, sprains) (527/983; 53.6%), followed by head injuries (238/983; 24.5%), limb fractures (166/983; 16.9%) and trunk injuries (52/983; 5.3%). However, a few patients sustained severe injuries, including skull fractures (7/238; 2.9%), intracranial haematoma (4/238; 1.7%) or lacerations of abdominal organs (4/52; 7.7%). This study presented a consistently high frequency of scooter injuries in children. Children under 15 years were the most affected by scooter-related injuries. Although most injuries were minor, serious injuries occurred that should not be underestimated. Hence, we emphasise the use of protection gear and recommend raising awareness among parents and children.

## 1. Introduction

Nonelectric scooters (further referred to as scooters) appeared in the early 2000s. As a popular mode of transportation, the scooter was originally designed and planned for people in their early twenties [1]. A scooter consists of two small wheels, which are connected by a narrow footplate and a t-shaped handlebar and is easily portable [1]. Scooters can reach a speed of 8 to 10 kilometres per hour, depending on the strength and weight of the rider and the slope of the road [2]. This achieves a speed similar to that of inline skates or skateboards [1]. The popularity of the scooter is evidenced by an increase in sales, which was accompanied by an increase in scooter-related injuries in the early 2000s [1,3,4]. This increase was recorded in the national registers, which contain mainly minor injuries but also deaths [5]. 

Although scooters were intended for young people, these vehicles are mostly used by children under 15 years [1,6,7]. This fact is reflected in the NEISS data of 2000: 85% of children with scooter-related injuries were younger than 15 years of age, and 30% of those children were even younger than 8 years of age [2]. Children and young people use these scooters not only as a means of transport but also for playing and recreational activities, such as performing tricks in the park [1,8]. It is described that many accidents occur on uneven ground at low speed because the small wheels of the scooter become stuck. In addition, younger children are more prone to falls because of being unable to keep their balance [2,9]. Further, known mechanisms for paediatric scooter-related injuries are that children and young people collide with other objects or road users or fall and get injured while trying out risky tricks in the park [1,8]. The pattern of these scooter-related injuries are similar to skateboard- and bicycle-related injuries in children, including the majority of upper limb fractures and minor injuries of the head [5]. 

The World Health Organisation (WHO) supports improving and ensuring the quality and quantity of data on childhood injury morbidity and outcomes in order to target investments in injury prevention [10]. 

A main topic of scooter injuries already studied in the early 2000s was the availability of prevention measures and protective equipment and whether or not they were used [1,2,5,9,11].

Protective devices, such as wrist guards, knee pads and helmets, were already in place and used by skateboarders and cyclists to reduce the risk of wrist, lower limb fractures and head injuries, which also affect scooter riders [12,13,14,15]. In addition, helmets have been shown to reduce concussion symptoms in scooter-related head injuries, which, according to the literature, are the least severe of all recreational vehicle-related head injuries [16,17]. The literature also shows that legislation requiring helmets to be worn is an effective means of reducing injuries associated with recreational vehicles and is still an area for intervention [13]. 

In addition, the literature reports that although up to 70% of parents and children know about and have access to safety equipment, only a minority (16%) actually use it [9]. Data on the use of protective clothing vary from “only one patient” to “seldom” and only a minority and ranges from 3% to 16% of patients [1,2,5,8]. 

In general, children seem to be less likely to use protective equipment when riding a scooter than in any other type of activity studied [1]. In addition, the data suggest that children often ride scooters without the supervision of a parent or adult [2]. 

If prevention mechanisms had been introduced, an overall decrease would have been expected during the observation period.

Nowadays, electric scooters have become the focus of epidemiological studies, but an increasing number of incidents of injuries on nonelectric scooters is still of interest due to their continued popularity, and the question is whether there are changes in frequency and behaviour.

The aim of this study was to illustrate the frequency of scooter-related injuries by giving an epidemiological overview of injuries during the last eight years.

## 2. Materials and Methods

This epidemiological, retrospective study was approved by the Ethics Committee of the Medical University of Vienna (Code: 1178/2023) on 14 March 2023 and according to the Declaration of Helsinki in its latest amendment. 

In total, 983 children and adolescents aged from 0 to 18 years were treated after nonelectric-scooter-related injuries at the Department of Trauma Surgery at the Medical University of Vienna from 2015 to 2022.

Data were conducted retrospectively by reviewing all patient charts of children and adolescents involved in scooter-related incidents, including the following: age, sex, injury mechanism (“fall/crash” or “traffic”), alcoholic abuse, and diagnosed injuries. Falls/crashes were defined as injuries after falling off the scooter from a standing position or through colliding with another object and a subsequent fall. Traffic accidents were defined as accidents in which patients collided with other road users, such as pedestrians, scooter riders, cyclists, cars or other means of public transport. The observed injuries were classified and categorised as follows into head injuries (contusion, concussion, skull fracture and intracranial haematoma), epiphysiolysis and fractures of the upper and lower limbs, injuries of the trunk (osseous contusion including thoracic and vertebral contusions, fractures of the thorax or vertebrae, abdominal contusion, lacerations of the abdominal or thoracic organs) and minor injuries (contusions, sprains, wounds and others). 

For head injuries, the following additional data were collected: radiological diagnostic device (X-ray or computer tomography scan (CT scan)). Moreover, the type of treatment, hospital stay in days, follow-up (FUP) in days and deficits at the last FUP were documented for all patients.

All patients with minor injuries were only treated on an outpatient basis, and wound checks were outsourced to the primary care physician to relieve the system.

Descriptive analysis (mean, range and standard deviation) was performed for the entire patient cohort. In order to provide an epidemiological overview, the following parameters were included: age, sex, injury mechanism, diagnosis, alcoholic abuse, radiological diagnostic device (X-ray or CT scan), treatment (conservative or surgical), hospital stay in days, FUP in days and deficits at the last FUP.

For metric variables (age or FUP in days), mean values and standard deviation values and for categorial variables (injury mechanism, injuries and type of treatment), frequencies and percentages were determined. 

All statistical analyses were performed using Microsoft^®^ Excel macOS software (Version 16.42 Microsoft Corp., Redmond, WA, USA) and SPSS^®^ software (Version 27.0.0., SPSS Inc.: Chicago, IL, USA).

## 3. Results

The total study population consisted of 983 (mean age: 7.9 ± 4.0 years; 383 female (f), 39.0%, mean age: 6.0 ± 5.7 years; 600 male (m), 61.0%, mean age: 10.0 ± 2.8 years) children and adolescents (Table 1). 

The frequency of scooter injuries was relatively consistent over the observation period. Minimal fluctuations were present, with a minimum of 86/983 (8.7%) scooter-related injuries in 2021 and a maximum of 157/983 (16.0%) injuries in the following year, 2022, as illustrated in (Table 2). 

### 3.1. Causes of Accidents

In total, 958/983 patients (97.5%; mean age: 7.9 ± 4.0 years) were injured after falling off their scooter, and 25/983 (2.5%; mean age: 9.0 ± 3.6 years) were injured in traffic accidents (Table 3). Four of these twenty-five (16.0%) children were seriously injured: two children sustained fractures of the lower limbs, including a bimalleolar fracture and an open fracture of the lower leg. Both fractures indicated surgical treatment, and these children had to stay in hospital (mean inpatient stay, 5 ± 4.2 days). The other two children sustained severe head injuries, including an open head fracture with subarachnoid haematoma and a basilar skull fracture with intracerebral haematoma. The patient with the basilar skull fracture was surgically treated, and both patients had an inpatient stay of 11 days. In addition, 40% of patients (10/25) involved in traffic accidents suffered head injuries, including seven contusions of the skull, one concussion, one open skull fracture and one intracranial bleeding combined with a skull base fracture. 

### 3.2. Seasonal and Diurnal Distribution of Accidents with Scooters

**Figure 1:**
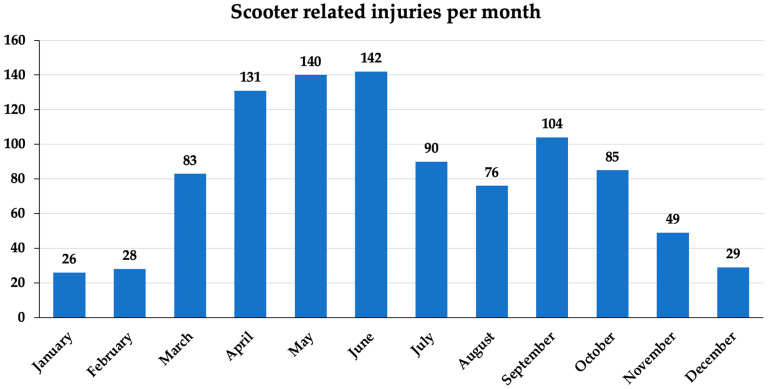
Frequency of injuries on scooters per month for the entire observation period of 8 years, with most injuries occurring in spring months (April–June).

Most accidents occurred from April to June, with a mean of 138 ± 5.9 patients sustaining injuries on scooters each month. Most accidents occurred in June, with 142 patients injured (Figure 1).

It appeared that most scooter accidents occurred during the day, mostly between 4pm and 9pm, with 422 patients (58.3%). Accidents during night-time were low, with 21 patients (2.9%) injured between 10p.m. and 3a.m. (Table 3). The time of accident was documented in only 724/983 patients.

### 3.3. Head Injuries

In total, 238/983 patients (24.5%; mean age: 6.6 ± 4.0 years; 86 f, 151 m) sustained head injuries. These observed head injuries included contusions (205/238, 86.1%), concussions (22/238, 9.2%), skull fractures (7/238, 2.9%) and intracranial haematoma (4/238, 1.7%).

In total, 12/238 (5.0%) patients with head injuries had a CT scan after a primary X-ray, whereas 212/238 (89.1%) patients received only an X-ray. In 14/238 (5.9%) patients, radiological examination was omitted due to missing external signs of injury. In total, 3/238 (1.3%) patients (1x skull fracture, 2x intracranial haematoma) required surgical treatment. In total, 44/238 (14.3%) patients stayed in hospital. Children and adolescents who sustained a concussion or contusion of the skull remained for 1.0 ± 0.9 days for observation, whereas patients with skull fractures or intracranial haematoma remained for 7.0 ± 3.0 days.

### 3.4. Fractures of the Upper and Lower Limbs

In total, 166/983 patients (16.9%; mean age 9.6 ± 3.1 years, 71 f, 95 m) sustained limb fractures, and 19/166 (11.4%) were treated surgically. 

A total of 124/166 (74.7%; mean age: 9.8 ± 3.2 years) sustained upper limb fractures, occurring mostly in the distal arm (56/124; 45.2%). In total, 12/124 patients (9.7%) received surgical treatment and stayed in hospital for 1.6 ± 0.7 days and had a mean FUP of 79.8 ± 50.4 days.

A total of 42/166 patients (25.3%; mean age: 8.8 ± 2.9 years) sustained lower limb fractures, mostly in the lower leg, especially isolated tibia fractures. Thirty-five of the lower limb fractures were treated conservatively. Seven patients were surgically treated and stayed in hospital for 4.4 ± 2.8 days and had a mean FUP of 222.0 ± 183.3 days. 

### 3.5. Trunk Injuries

A total of 52/983 patients (5.3%; mean age: 8.4 ± 3.7; 19 f, 34 m) sustained trunk injuries, mostly osseous contusions (22/52, 42.3%), followed by abdominal contusions (15/52, 28.8%), fractures including clavicle and vertebral fractures (7/52, 13.5%), and sprains (4/52, 7.7%). Four patients sustained laceration of abdominal organs without needing surgical intervention. The mean hospital stay was 2.6 ± 3.2 days. 

### 3.6. Minor Injuries and Other

In total, 527/983 patients (53.6%; mean age: 8.0 ± 4.1 years; 279 f; 319 m) sustained minor injuries, including contusions (219/527, 41.6%), sprains (40/527, 7.6%), wounds or excoriations (213/527, 40.4%) or others (55/527, 10.4%). 

One 8-year-old patient, who was involved in a traffic accident, sustained a polytrauma, including an intracranial haematoma, skull base fracture, multiple contusions and wounds. This patient received surgical treatment. 

No persistent deficits or symptoms were conducted, and the mortality in this study was 0%.

## 4. Discussion

The literature states that scooters were and are still very popular, as evidenced by the continuing high sales figures and the increase in injuries caused by scooters [1,3,18]. The continued popularity of scooters is also reflected in this study by the consistently high number of scooter-related injuries over the last 8 years, despite the known risk of injury from riding scooters [8,19]. The data suggest that parents and children should continue to be informed about the risks associated with riding scooters.

During this eight-year observation period, the number of injuries was lowest in 2021, followed by an absolute peak in 2022. The reasons for this exceptional low in 2021 remain unclear. Interestingly, the number of scooter injuries remained constant during the strict lockdowns in 2020 during the COVID-19 pandemic, which is consistent with the observation of relatively constant injury patterns during lockdowns [20]. During the strict lockdown in March 2020, 10 patients presented with scooter-related injuries, which is also the mean frequency (10.4 ± 3.4 patients) in the month of March. The other lockdown months (November and December) also show no difference compared to the mean frequencies, suggesting that the COVID-19 pandemic did not lead to a reduction in scooter-related injuries as might have been assumed due to the fact that children and adolescents had to stay at home and parks were closed. 

The majority of the study population (600 patients; 61.0%) was male. This, and the fact that the male patients were generally older than the girls (mean age: 10.0 ± 2.8 years vs. 6.0 ± 5.7 years), is in line with the results of previous studies, and it can only be assumed that boys play wilder than girls [1,2,9]. Regarding the age difference between girls and boys, it can only be speculated that girls’ interest in riding scooters decreases with age or that boys take more risks with age, leading to a higher frequency of scooter-related injuries. The vast majority of patients (958; 97.5%) were injured in a fall from a scooter from a standing position without being involved in a traffic accident. The rarity of traffic accidents (25; 2.5%) is within the range in the literature (up to 10%) [2,6,18] and could be related to the fact that scooters are more likely to be used by younger children riding in parks [1,2,6,9], which is also reflected in this study, with a mean age of 7.9 ± 4.0 years [1,2,19]. This suggests that scooters are primarily used as a toy rather than a means of transport as opposed to electric scooters. This might be supported by the occurrence of scooter-related injuries primarily during spring and summer months, with a maximum of 142 injuries in June in this study, which is similar to the literature [2,19,21]. In addition, a decrease in injuries was noted in July and August, which may be related to the fact that children and their families were on vacation during these months. Further, most injuries occurred in the late afternoon and evening hours between 4 and 9 p.m. (422; 42.9%). 

Although most children and adolescents suffered only minor injuries (527; 53.6%), there were isolated cases of severe injuries (181/983; 18.4%), of which 23/181 (12.7%) required surgery. The frequency of head injury in this study was 24.5%, comparable to the range reported in the literature (8–33% head injuries) [1,2,5,9,19,22]. However, a study by Mankovsky et al. presented a higher frequency of head injuries (up to 47.5%) because, unlike in this study, facial injuries, such as wounds, were also included [4]. The distribution of fractures was similar to that reported in the literature, with the upper limbs being the most commonly affected (124/166; 74.7%), particularly the distal forearm (57/124), reflecting the usual cause, which was a fall on the outstretched arm [5,8,9]. The frequency of lower limb fractures (42) and trunk injuries (52) was also in line with the literature [18]. Severe trunk injuries (four lacerations of the spleen or liver) were rare and seen when patients were struck in the abdomen by the handlebars of the scooter during a fall, comparable to the literature [4,23]. 

In addition, this study shows that the injury patterns in scooter accidents are similar to accidents involving skateboards or bicycles when compared to the existing literature, as falling on the outstretched hand or the head was the most common [1]. In this context, studies showed a decrease in injuries by using wrist protection gear in snowboarding or inline skating [24,25]; therefore, the use of wrist protection gear may also be beneficial for scooter riders. Especially, children doing tricks in parks and on ramps should be instructed to wear wrist protection like skateboarders. 

In addition, studies state that although up to 70% of children, adolescents and parents know about and have access to safety equipment, only a minority (16%) actually use it [1,9,26]. Wearing protection gear for the wrist is supposed to decrease fractures of the distal arm, as this is a common fracture localisation of the upper limb (57/124). Further, it is described that the use of helmets decreases the frequency of head injuries while riding bicycles or scooters [13,14,15]. In addition, helmets have also been shown to reduce concussion symptoms in scooter-related head injuries, which, according to the literature, are the least severe of all recreational vehicle-related head injuries [16,17]. Although most head injuries in this study were minor, including contusions of the head (205/238, 86.1%), concussions (22/238, 9.2%), intracranial haematoma (4/238, 1.7%) and skull fractures (7/238, 2.9%) were also documented. As protective measures, such as wrist gear and helmets, already exist and have been proven to decrease the risk of injury in other studies [12,13,14,15], parents need to be made aware to use them. Further, in this study, 40% (10/25) of children and adolescents suffered head injuries when involved in traffic accidents; therefore, the use of helmets in public should be more strictly controlled. 

Generally, it can be assumed that due to the relatively constant number of scooter injuries during the entire observation period, prevention strategies are still not yet fully implemented and could yet be improved, or even further measures added.

Overall, it can be hypothesised that if such preventive measures were consistently applied for children and adolescents, the frequency of scooter-related injuries might decrease. Such measures could include that children and adolescents, together with their parents, have to be made aware of the risks and potential danger of riding scooters in public, have to be introduced to various prevention strategies, and scooter riders should be encouraged to wear protection gear. Suggestions could include advertisements placed in public, at public events and on social media, television, etc. Another suggestion would be to distribute information brochures and show informative short films in the waiting areas of trauma units. However, the suggestions for advertising and the distribution of information material would need to be further explored to determine whether such measures would be effective and, therefore, justified. One suggestion that seems to be effective and helpful is the introduction of compulsory helmet use by law. The literature shows that legislation requiring helmets to be worn is an effective means of reducing injuries associated with recreational vehicles and is still an area for intervention [13]. 

In addition, adult supervision seems to be a further important factor due to the young age of patients that could help reduce these injuries. Similar to the literature, 920/983 (93.6%) patients were younger than 15 years and 461/983 (46.9%) even under 8 years [2,27]. The studies state that children often sustained scooter-related injuries when adult supervision was missing [1,2,8]. Furthermore, Fong et al. state that supervising adults do not emphasise wearing protection gear enough, reflecting that parents underestimate the risk of scooter accidents and the resulting injuries [8]. Regarding these facts and the young mean age of this study, it is highly recommended to educate parents concerning safety strategies in kindergarten and preschool within the framework of regular discussions with educators or the implementation of an educational “Safety-Day” once a year in order to decrease scooter-related injuries in the future.

### Limitations

This study is limited by its retrospective single centre design. However, this study was conducted at a Level 1 trauma centre, and due to the long observation period, the study population (*n* = 983) can be considered large compared to the literature [1,2,8,9]. Most study populations consisted of less than 100 patients over an observation period of one or two years. This may be related to the fact that these studies [1,2,8,9] were conducted in the early 2000s when scooters became popular. Thus, this study presents a relatively steady number of scooter injuries, with an increasing tendency in 2022 compared to previous years. Considering the longer observation period, this study is one of few presenting the course over the last years, whereas most studies have a relatively short observation period of only one to two years [1,2,8,9]. An increasing tendency was also shown by Unkuri et al., presenting seven patients in 2008 compared to fifty-five patients in 2015 [18]. Recent studies mostly focus on injuries associated with electric scooters, so this study is distinguished by providing current data on nonelectric scooters only [28,29,30]. As the numbers of scooter-related injuries are still high and do not tend to decrease, it is especially important to educate parents, children and adolescents about the risks and how to prevent them.

Unfortunately, due to the incomplete documentation of worn protection gear in this study, no direct conclusions about the benefits of wearing protection gear can be made based on these data. Indirectly, it can be assumed that protection gear was barely used because of the relatively constant frequency of injuries, particularly the frequency of fractures. However, based on the literature, it has to be assumed that also the relatively high frequency of head injuries and wrist fractures due to scooter injuries could be decreased by actually wearing diverse means of protective gear [13,14,31]. Thus, it has to be emphasised that based on the constant frequencies, it is recommended to establish wearing protective gear for children, plus educating parents about the mechanisms and risk of scooter-related injuries, to reduce the number of these injuries in the near future.

## 5. Conclusions

Considering the background population of over 332,000 children and adolescents in the relevant age group in Vienna, Austria, and the 8-year period of data collection in primary care, scooter riding appears to be a relatively safe means of increasing physical activity in children and adolescents. During this observation period, the average number of injuries per year in our trauma unit was 9300, with an average of 8439 children and adolescents, which means that on average 1.5% of all children sustained a scooter-related injury per year. Nevertheless, this study shows that the number of scooter-related injuries in children and adolescents remained relatively steady during the observation period and had an increasing tendency. Children under the age of 15 were usually the most affected by scooter injuries. Although most injuries were minor, serious injuries occurred that should not be underestimated. Given these data, the use of protective equipment by children and the education of parents is still highly recommended and has yet to be implemented to prevent and reduce scooter-related injuries in the near future.

## Figures and Tables

**Table 1:** Gender-specific overview of the total cohort plus mean age.

	Total	%	Mean Age
Female	383	39.0	6.0 ± 5.7
Male	600	61.0	10.0 ± 2.8
Total	983	100	7.9 ± 4.0

**Table 2:** Frequency of paediatric scooter-related injuries from 2015 to 2022.

Scooter Injuries		2015	2016	2017	2018	2019	2020	2021	2022
In total	983	130	119	102	131	133	125	86	157
%	100	13.2	12.1	10.4	13.3	13.5	12.7	8.7	16.0

**Table 3:** Descriptive analysis of the total cohort.

	Number of Patients	%	Mean Age in Years
Total	983	100	7.9 ± 4.0
Fall	958/983	97.5	7.9 ± 4.0
Traffic accident	25/983	2.5	9.0 ± 3.6
			
4 a.m. to 9 a.m.	58/724	8.0	
10 a.m. to 3 p.m.	223/724	30.8	
4 p.m. to 9 p.m.	422/724	58.3	
10 p.m. to 3 a.m.	21/724	2.9	
			
Head injuries	238/983	24.5	6.6 ± 4.0
Contusion	205/238	86.1	
Concussion	22/238	9.2	
Skull fracture	7/238	2.9	
Intracranial haematoma	4/238	1.7	
			
Fractures	166/983	16.9	9.6 ± 3.1
Upper limb	124/166	74.7	
Lower limb	42/166	25.3	
Surgery	19/166	11.4	
Conservative treatment	147/166	88.6	
			
Trunk injuries	52/983	5.3	8.4 ± 3.7
Osseous contusion	22/52	42.3	
Abdominal contusion	15/52	28.8	
Fracture	7/52	13.4	
Sprain	4/52	7.7	
Organ laceration	4/52	7.7	
			
Minor injuries	527/983	53.6	8.0 ± 4.1

## Data Availability

The datasets generated and/or analysed in the current study are not publicly available due to data privacy but are available from the corresponding author on reasonable request.
